# Force-Sensitive Mat for Vertical Jump Measurement to Assess Lower Limb Strength: Validity and Reliability Study

**DOI:** 10.2196/27336

**Published:** 2021-04-09

**Authors:** Erik Vanegas, Yolocuauhtli Salazar, Raúl Igual, Inmaculada Plaza

**Affiliations:** 1 Electrical/Electronics Engineering and Communications Department, EUP Teruel Universidad de Zaragoza Teruel Spain; 2 Tecnológico Nacional de México IT Durango Durango Mexico

**Keywords:** vertical jump, mHealth, mobile health, force-sensitive resistor, lower limb strength, leg strength

## Abstract

**Background:**

Vertical jump height is widely used in health care and sports fields to assess muscle strength and power from lower limb muscle groups. Different approaches have been proposed for vertical jump height measurement. Some commonly used approaches need no sensor at all; however, these methods tend to overestimate the height reached by the subjects. There are also novel systems using different kind of sensors like force-sensitive resistors, capacitive sensors, and inertial measurement units, among others, to achieve more accurate measurements.

**Objective:**

The objective of this study is twofold. The first objective is to validate the functioning of a developed low-cost system able to measure vertical jump height. The second objective is to assess the effects on obtained measurements when the sampling frequency of the system is modified.

**Methods:**

The system developed in this study consists of a matrix of force-sensitive resistor sensors embedded in a mat with electronics that allow a full scan of the mat. This mat detects pressure exerted on it. The system calculates the jump height by using the flight-time formula, and the result is sent through Bluetooth to any mobile device or PC. Two different experiments were performed. In the first experiment, a total of 38 volunteers participated with the objective of validating the performance of the system against a high-speed camera used as reference (120 fps). In the second experiment, a total of 15 volunteers participated. Raw data were obtained in order to assess the effects of different sampling frequencies on the performance of the system with the same reference device. Different sampling frequencies were obtained by performing offline downsampling of the raw data. In both experiments, countermovement jump and countermovement jump with arm swing techniques were performed.

**Results:**

In the first experiment an overall mean relative error (MRE) of 1.98% and a mean absolute error of 0.38 cm were obtained. Bland-Altman and correlation analyses were performed, obtaining a coefficient of determination equal to *R*^2^=.996. In the second experiment, sampling frequencies of 200 Hz, 100 Hz, and 66.6 Hz show similar performance with MRE below 3%. Slower sampling frequencies show an exponential increase in MRE. On both experiments, when dividing jump trials in different heights reached, a decrease in MRE with higher height trials suggests that the precision of the proposed system increases as height reached increases.

**Conclusions:**

In the first experiment, we concluded that results between the proposed system and the reference are systematically the same. In the second experiment, the relevance of a sufficiently high sampling frequency is emphasized, especially for jump trials whose height is below 10 cm. For trials with heights above 30 cm, MRE decreases in general for all sampling frequencies, suggesting that at higher heights reached, the impact of high sampling frequencies is lesser.

## Introduction

Vertical jump height is one of the physical skills commonly used to assess overall performance in human beings, and more specifically, it is used to assess performance and muscle power of the quadriceps, hamstrings, and gastrocnemius muscle groups in the lower limbs [1,2]. Measurement of the performance of this skill is commonly performed on athletes in sports like basketball [3,4], football [5], netball [6], swimming [7], and others. This skill performance can also provide important data from people with no relevant sports past.

In the literature, there are many protocols to prove or validate the proposed systems. Among the different kind of jumps performed in those protocols, there are jumps with and without countermovement [1,4,5,8-15], jumps with and without arm swing [12,16], drop jumps [1,8,17], single and double leg jump [6], continuous jumps [4,17], squat jumps [1,2,4,12], and loaded squat jumps [7]. With any of these types of jumps, height reached by the user can be analyzed, but the jumps most commonly used in all related work are the countermovement and squat jumps.

Kibele [15] and Moir [18] reported that irregularities detected in measurements of any kind of jump execution may be linked to changes in the posture of a subject during flight due to change in the center of mass of the subject during the jump. Bui et al [13] found that some common errors obtained during measurement were caused by body movements like knee, hip, and ankle bending during flight time and landing. Also, Aragon-Vargas [19] states that ascending and descending phases of flight time must be of the same length of time, but in his work descending time was significantly longer, suggesting that participants descended with their bodies partially crouched.

There are several techniques to measure jump height, each of which uses a different kind of sensor or no sensors at all. Methods like the Sargent jump [13] and Vertec device [6,9,10] require no sensors and often are used as reference measurements. However, these methods often show overestimation on jump height, and this could be due to arm stretching performed unconsciously by the user. Among systems developed in the literature, different kind of sensors are used like force-sensitive resistors (FSRs) [3,16], capacitive sensors [5], inertial measurement units [2,4,8,10,17], electromyography sensors [1,6], kinematic sensors [6], ultrasonic sensors [20], microswitches [9], video cameras, [11] and optical sensors [4,12].

The studies of Drazan et al [3] and Boukhenous and Attari [16] are most closely related to our work, as both of their systems also use resistive sensors. However, only one sensor is used for the whole sensing area. Drazan et al [3] proposed a system based on a single FSR sensor whose total sensing area is around 3 cm^2^, with an Arduino board as microcontroller. This system calculated jump height through the flight-time formula, by measuring the time the FSR sensor is not detecting any pressure. In the work presented by Boukhenous and Attari [16], two metallic strain gauges were placed in the center of a rigid platform to measure the force applied by the ground. In this case, vertical ground reaction forces were used to calculate jump height. Rico et al [2] used pressure sensors located at the forefoot of the user to calculate flight time during vertical jump and compare it with data obtained from an inertial measurement unit system. However, few or no specific number of subjects are used in these studies, and no specific protocols or jump trials are performed. Also, no reference systems are used to compare the performance of the developed systems.

Camera-based systems are used as reference systems or as proposed methods to validate. In some studies, the famous motion capture system commonly used in videogames is used as a reference device [5,17,21]. Other studies use a similar method by tracking body markers placed strategically on the body [4,14,21]. Balsalobre-Fernandez et al [11,22] have analyzed the effectiveness and reliability of high-speed cameras as methods to estimate vertical jump height. In these studies, flight time of the subject is calculated by selecting the takeoff and landing frames of the recorded videos of jump trials, and, by applying the flight-time formula, jump height is obtained [3,8,9,11,14,16,22].

Only a few studies perform a validation with a relatively high number of subjects. Some studies that fulfill this criterion are the ones presented by Nuzzo et al [10], Casartelli et al [4], Glatthorn et al [12], Moir [18], and Aragon-Vargas [19]. However, these studies compare different commercially available devices (contact mat, force plate, cameras, etc), and no novel system is developed by the researchers. Bui et al [13] fulfills the criterion and proposes a novel optical system whose performance is compared against commercial devices. This system calculates jump height through the flight-time formula.

This study presents a newly developed low-cost system for measuring height reached by users during vertical jump comprising a matrix of FSR sensors embedded on a mat. The height of the vertical jump is calculated through the flight-time formula [3,8,9,14,16]. One advantage this system offers against other pressure-sensitive systems [2,3,16] is a higher real sensing area, higher resolution, and higher precision, as this system works with 256 FSR sensors distributed around the mat in 16 rows and 16 columns, in comparison with the other systems that use a single sensor. The total sensing area, dimensions of FSR sensors matrix, and each individual FSR sensor area can be modified on different versions of the proposed mat. Also, as this system is environmental, it needs no adjustment regardless of the physical characteristics of the user like body type, weight, height, or foot size [2]. Another advantage of this system is that the calculated vertical jump height is directly sent to a PC or mobile device of the health care professional’s choice, unlike other methods that require postprocessing analysis, as in the high-speed camera method. The main objective of this study is to validate the reliability of the proposed system for future clinical studies.

## Methods

### System Construction

The proposed system consists of 2 parts: a resistive pressure-sensitive mat constructed with an FSR sensor array and the electronic system. The mat is composed of 3 layers. One layer contains thin and flat copper wires distributed in a column arrangement along a flexible 3D printed grid; another layer is composed of the same copper wires but distributed in a row arrangement on another flexible 3D printed grid. A third layer is placed between the layers consisting of Velostat material, a pressure-sensitive material that behaves as a resistor whose value drops whenever pressure is exerted upon it. In this way, variations on the resistance values on every intersection of rows and columns when pressure is exerted over the mat can be measured. Some typical characteristic problems with Velostat material are repeatability, nonlinearity, and hysteresis [23,24]. For this application, however, these features are not relevant because high precision is not needed; the mat only needs to be able to detect a heavy body placed on it (average human body weight). More information about the development of this mat is documented on the work of Medrano et al [25].

**Figure 1:**
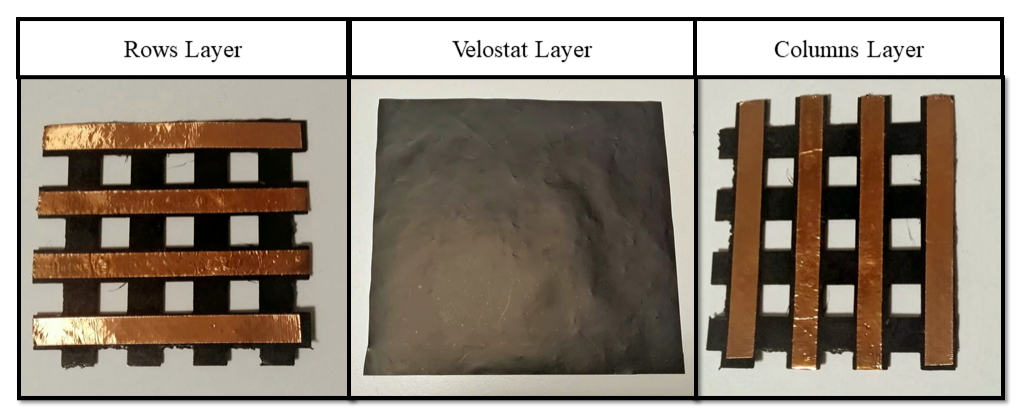
Different layers comprising a force-sensitive resistor matrix with smaller dimensions.

**Figure 2:**
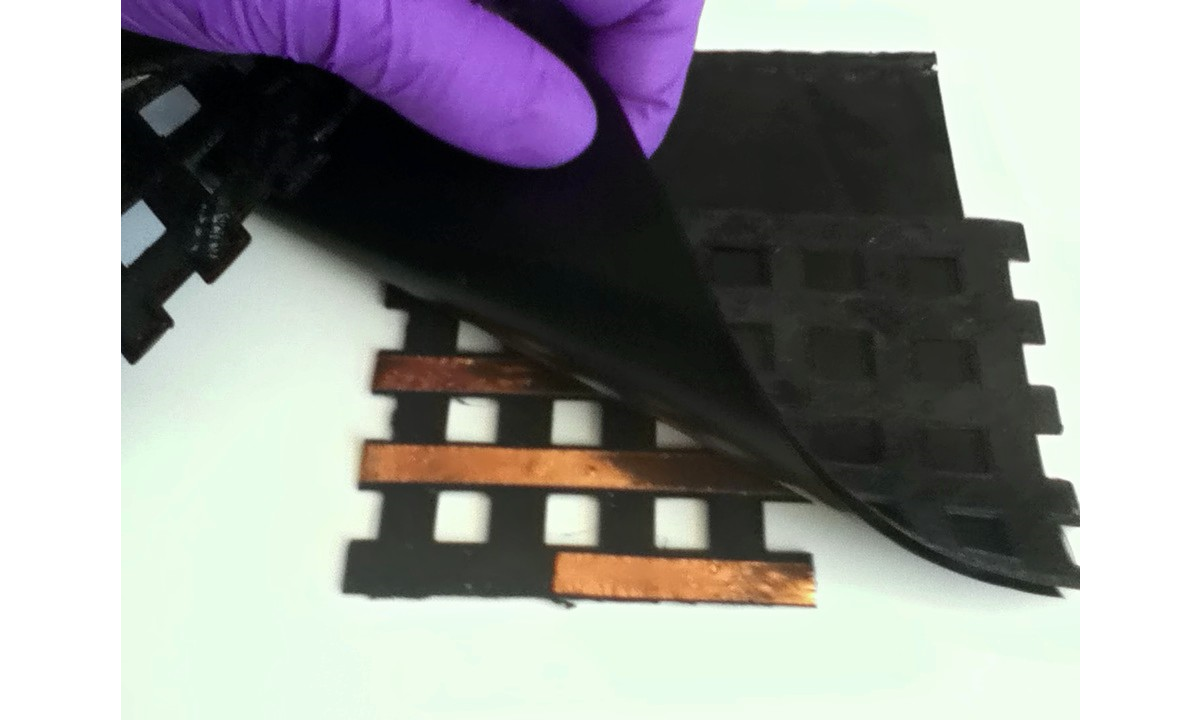
Example of a smaller size matrix and how layers are placed.

**Figure 3:**
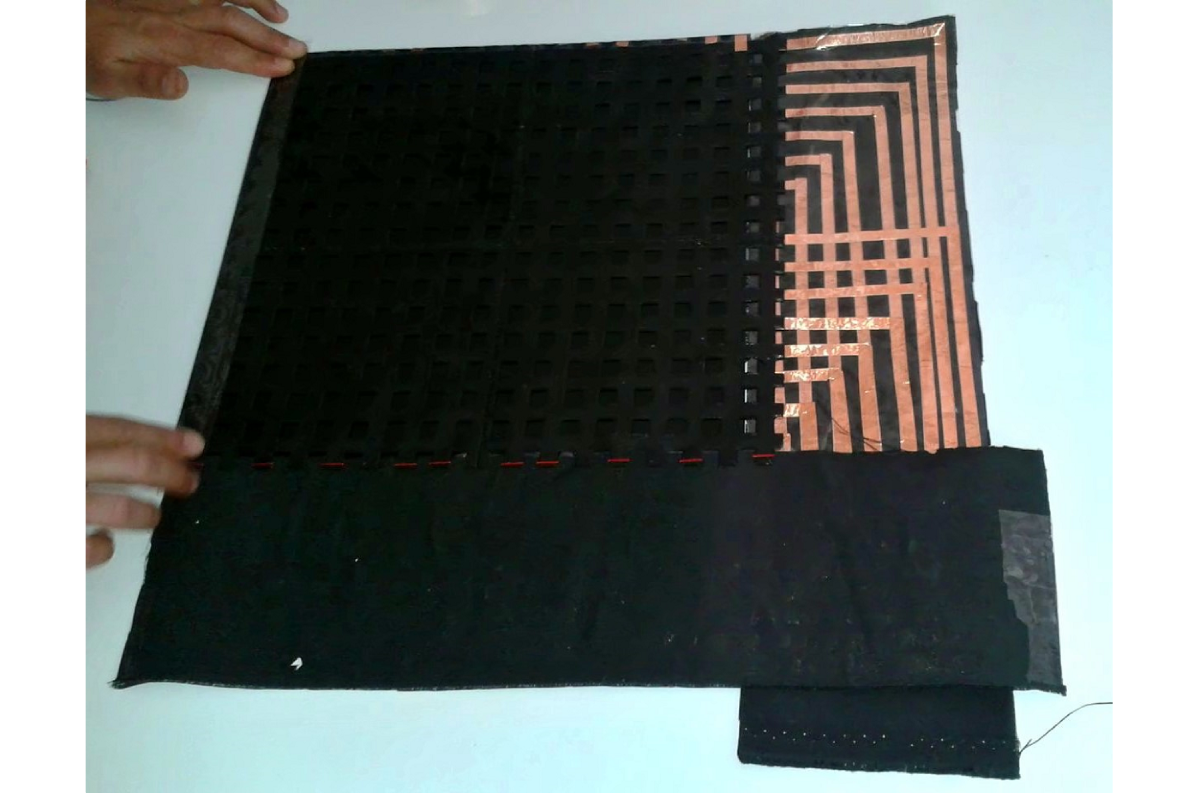
Developed force-sensitive resistor sensor mat.

Figure 1 shows the different layers of an FSR matrix with smaller dimensions (4 rows and 4 columns). Figure 2 shows an example of the placement of the overlapped layers. The total sensing area of the mat used for this study is 30×30 cm, with 16 rows and 16 columns, 1 cm width each. This way, the area of each of the individual FSR sensors is equal to 1 cm^2^. In Figure 3, a developed mat is shown.

**Figure 4:**
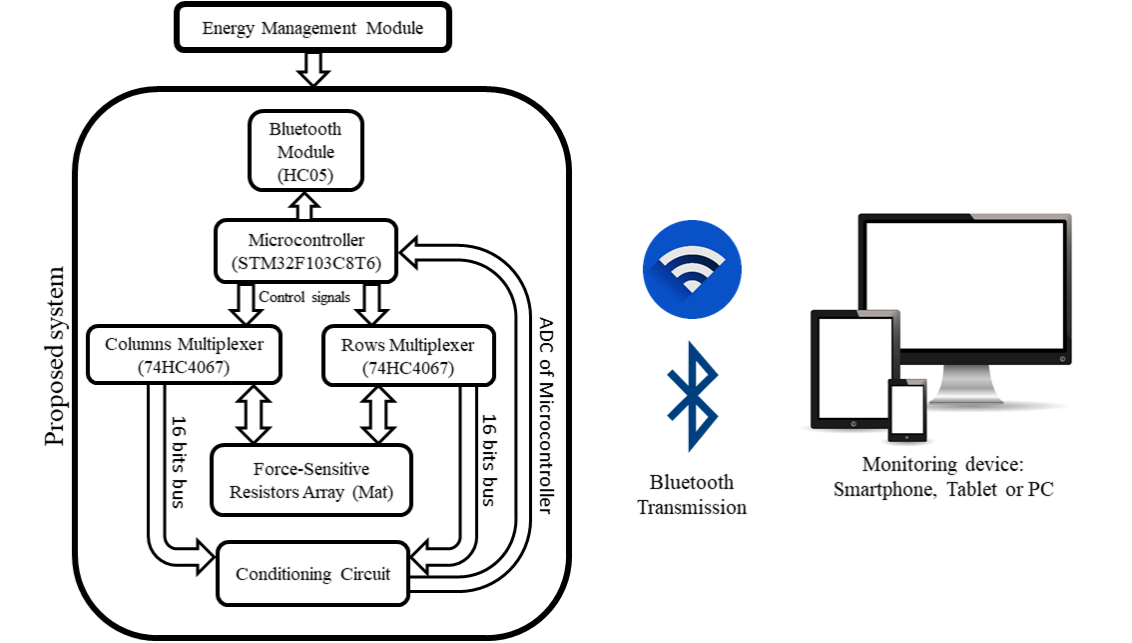
Block diagram of the proposed system.

Due to the number of operations needed for a full scan of the mat, a high frequency microprocessor must be used for data processing, as the time complexity of these operations grows in exponential order. The STM32F103C8T6 microprocessor (STMicroelectronics) was selected due to its 72 MHz CPU frequency, with which a sampling frequency of 200 Hz is achieved. Other microprocessors with lower CPU frequencies (like the ATmega328P, Microchip Technology Inc) would not achieve the desired sampling frequency. Also two 16-1 multiplexers 74HC4067 are needed for an efficient scan process of the whole mat. For data transmission, a Bluetooth HC-05 module is used. Bluetooth technology was selected due to its ease of connection with different devices, especially with smartphones and tablets, which offers health care professionals the choice of an easy-to-transport monitoring device. Other electronic elements included in the system are a TP4056 battery-charging module and a Lipo battery of 3.7 V and 150 mAh capacity, allowing continuous functioning of the system for up to 2 hrs. A block diagram of the proposed system is shown in Figure 4.

The algorithm used for this system consists of calculating the summation of every FSR sensor of the mat. For each FSR sensor, the voltage value obtained by the analog-to-digital converter of the microcontroller is given in bits (from 0 to 4095), and this resulting value is used for the calculations. A threshold is used for the system to decide whether a person is standing on the mat or not. To calculate an appropriate value for this threshold, data were collected from 16 volunteers (5 female and 11 male), with an average weight of 74.81 (SD 15.25) kg and foot size of 26.93 (SD 1.94) cm. The volunteers were asked to stand on the mat barefoot in 4 different positions: with both feet standing still and on their forefoot and with one foot standing still and on their forefoot. Maximum values of pressure exerted on FSR sensors were used as reference for normalization, and the minimum value for activation of FSR sensors was considered as no volunteer standing on the mat.

Using such criteria, on average when standing still over the mat with both feet subjects activated 71.66% of the FSR sensors, and when standing on their forefoot with both feet, 28.9% of the FSR sensors were activated. When standing still and on their forefoot with only one foot an activation of 40.37% and 18.40% of the FSR sensors was registered, respectively. The minimum value of FSR sensors activation is registered when standing on one foot on their forefoot, with a value of 12.09%. All results are summarized in Table 1. By taking these results into account, and if it is assumed that volunteers may land first with one foot on their forefoot after a jump, a proper threshold should be proposed below the minimum value of FSR sensors activation. For this study, a threshold of 9% of FSR sensors activation is used. This threshold was chosen to be at three-quarters between the zero FSR sensor activation and the minimum FSR sensor activation recorded, to avoid any misreading from mechanical oscillation. It is worth noting that the minimum recorded value from sensor activation is an outlier. In future studies, the possibility of adding a personalized threshold for every subject could be assessed.

**Table 1:** Normalized force-sensitive resistor sensors activation registered from volunteers standing at different positions on the mat; standing still and on their toes with both feet and standing still and on their toes with one foot.

Sensor activation	Both feet %		One foot %	
	Standing	Toes	Standing	Toes
Average	71.66	28.90	40.37	18.40
Maximum	100.00	43.31	61.45	27.00
Minimum	44.00	23.70	22.64	12.09

For every position sensor activation percentage, a correlation analysis with the weight and foot size of the volunteers was performed. Analysis suggests that sensor activation is only moderately impacted by the weight of volunteers, and foot size of volunteers has a low impact on sensor activation. In Table 2, correlation values for different positions analyzed on the mat are shown.

To calculate the height reached by the user during the vertical jump, when the subject jumps and there is no contact on the mat, the system counts the elapsed time until the subject lands on the mat again (flight time), and with the calculated time the flight-time formula is used [3,8,9,14,16] to predict the height reached. This formula is defined as: *Height* = *g*∆*t*^2^/8, where g is the constant value of gravity force g=9.81 m/s^2^ and Δt is the flight time obtained by the system. Once the height of the vertical jump is obtained, this value is wirelessly sent via Bluetooth to the monitoring device selected by the health care professional.

**Table 2:** Pearson correlation coefficient values (R values) for different positions analyzed, calculated for weight and foot size of volunteers.

Position		Weight	Foot size
**Both feet**			
	Standing	–.684	–.522
	Forefoot	–.411	–.241
**One foot**			
	Standing	–.447	–.397
	Forefoot	–.394	–.463

### Experimental Setup

Two different experiments were performed. The purpose of the first experiment, in which 38 volunteers participated, was to validate the reliability of the proposed system. For the second experiment, the objective was to compare the effects of different sampling frequencies when calculating the height reached on the jumps, and 15 volunteers participated. The protocol used for each experiment is the same. For the first experiment, data are directly processed by the microcontroller and the predicted value is sent to the selected monitoring station. In the second experiment, raw data are obtained to perform an offline downsampling to analyze the effects of different sampling frequencies on the predicted result.

Researchers asked for assistance from a sports and fitness center to recruit volunteers who attended the center regularly for physical training. Researchers visited this center with all the necessary equipment for the implementation of the protocols, and installed it in an area specified by the managers of the center. No specific physical attributes were required from the volunteers, as these characteristics should not affect the performance of the system. Researchers approached people at the center, explained the purpose of the study, and politely asked for their collaboration on the protocols if they were available at any given moment. Before starting any trial, volunteers were asked if they had any kind of injury that could affect their physical integrity when performing the protocol, and if so, the trials would not proceed. Every volunteer gave their written consent for the performance of the proposed protocol. The countermovement jump (CMJ) and countermovement jump with arm swing (CMJAS) techniques were selected for this protocol. These jumping techniques are commonly used as a measure to assess the overall force and explosive power of the lower body muscles on a person [26], and it is considered as the most reliable jumping test for this purpose [27]. By adding an arm swing to the CMJ, with the proper technique, the height reached by the person is increased around one-third and up to two-thirds [28-30], which increases the dynamic range of data obtained.

**Figure 5:**
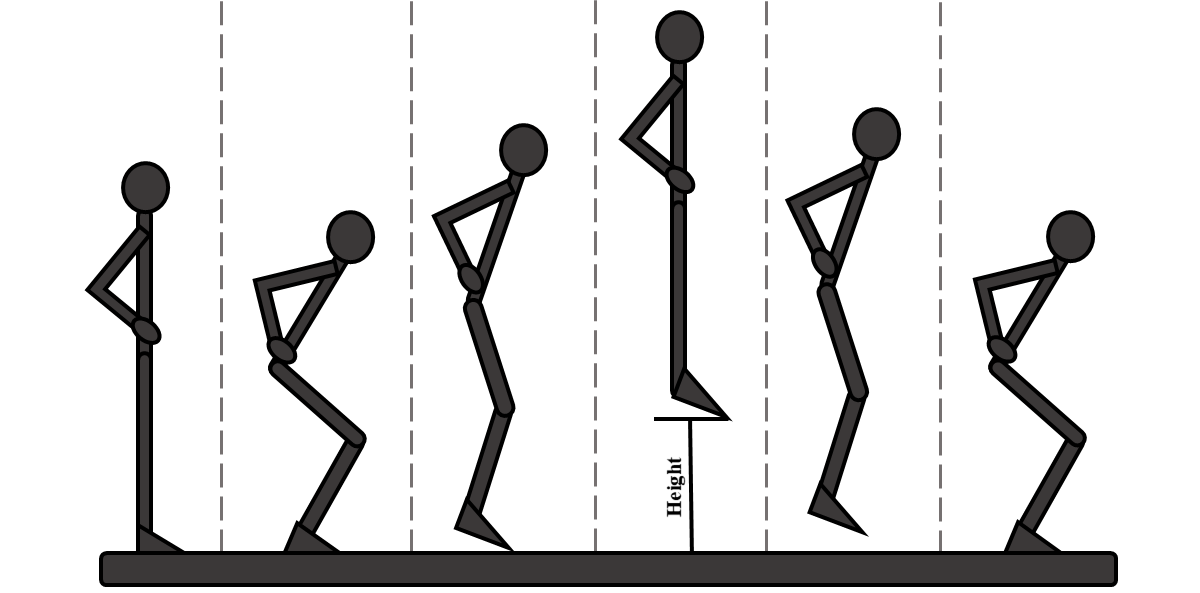
Countermovement jump technique, step by step.

**Figure 6:**
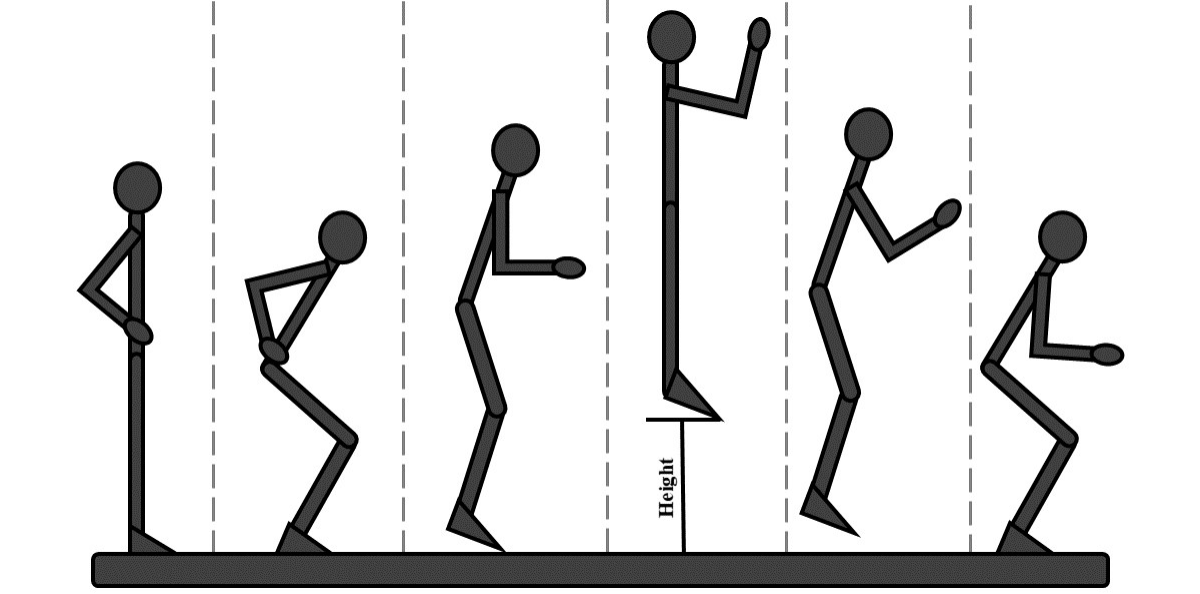
Countermovement jump with arm swing technique, step by step.

**Figure 7:**
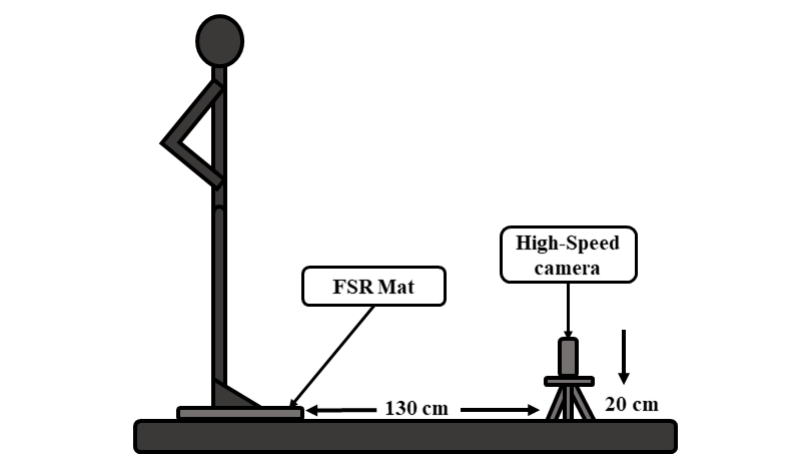
Experimental setup used for proposed experiments.

In the proposed protocol, volunteers were asked to stand on a marker placed on the center of the mat and perform 3 medium-to-maximal effort CMJs, with their hands fixed at the waist, with 5 to 10 seconds rest between trials. This technique is depicted in Figure 5. After these jumps, the volunteers were asked to perform CMJAS this time, and following the same scheme. This technique is depicted in Figure 6. As a reference system, all trials were recorded on video with a high-speed camera (120 fps). The camera was placed 1.3 m away from the mat, perpendicular to the sagittal plane of the volunteer and 20 cm above the ground, held by a tripod. The setup for this protocol is depicted in Figure 7.

To measure height reached by the subject with the video reference, the takeoff and landing frames were selected manually like in the studies of Balsalobre-Fernandez et al [11,22], and height was calculated by using the elapsed time between the frames using the flight-time formula.

## Results

### First Experiment: System Validation

**Figure 8:**
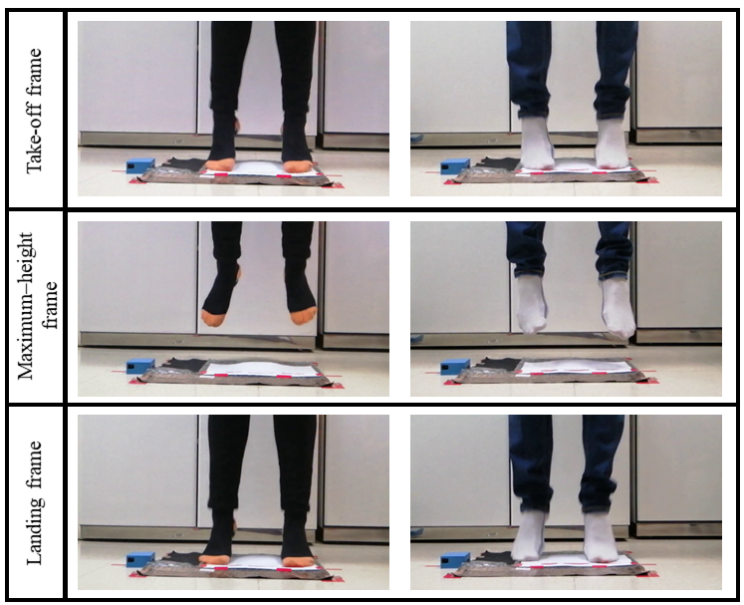
Two volunteers performing the proposed protocol showing the different phases of the jumps: takeoff frame, maximum-height frame, and landing frame.

For the first experiment, a total of 228 jumps (114 CMJs and 114 CMJASs) were performed for each, the proposed system and the video reference. An example of the recorded jumps is shown in Figure 8.

To analyze the proposed system performance, mean relative error (MRE) and mean absolute error (MAE) were calculated for the overall jump trials and for each technique, CMJ and CMJAS. The MRE obtained from all 228 trials was 1.98%. For CMJ and CMJAS, relative errors were 2.17% and 1.78%, respectively. MAE obtained from all jump trials was 0.38 cm, and for CMJ and CMJAS, the errors obtained were 0.34 cm and 0.42 cm, respectively. These results are summarized in Table 3.

**Table 3:** Mean absolute error and mean relative error values for overall jump trials, only countermovement jump, and only countermovement jump with arm swing trials.

Trials	MAE^a^ (cm)	MRE^b^ (%)
Overall	0.38	1.98
CMJ^c^	0.34	2.17
CMJAS^d^	0.42	1.78

^a^MAE: mean absolute error.

^b^MRE: mean relative error.

^c^CMJ: countermovement jump.

^d^CMJAS: countermovement jump with arm swing.

**Figure 9:**
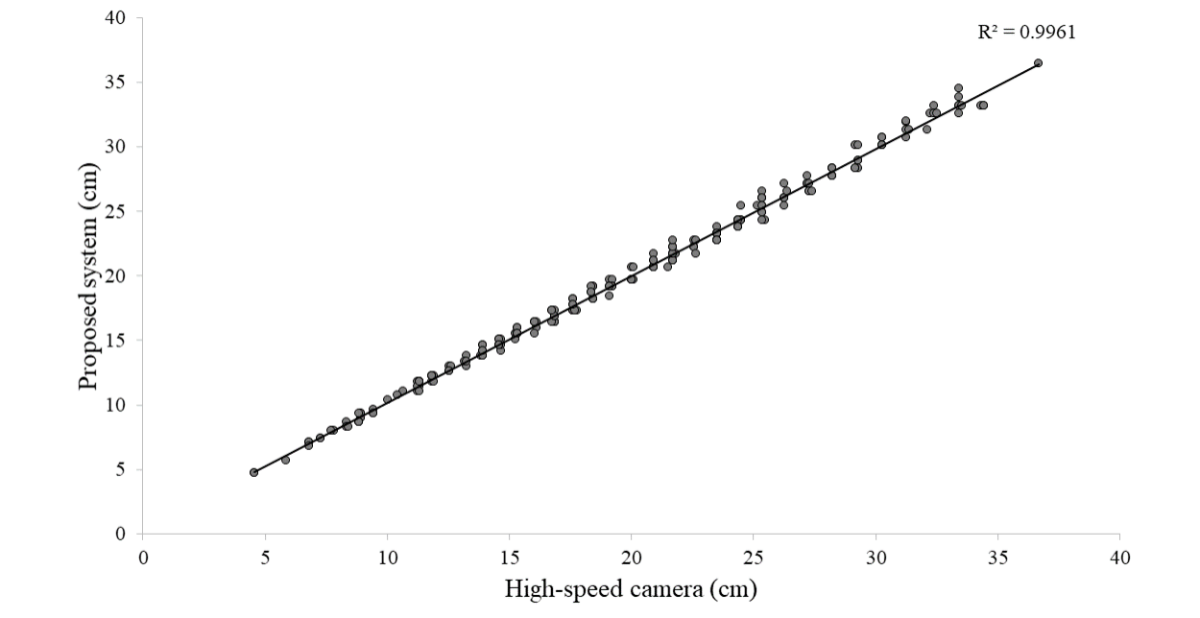
Correlation graph comparing both measuring methods for the first experiment, showing a coefficient of determination of *R*^2^=.996.

**Figure 10:**
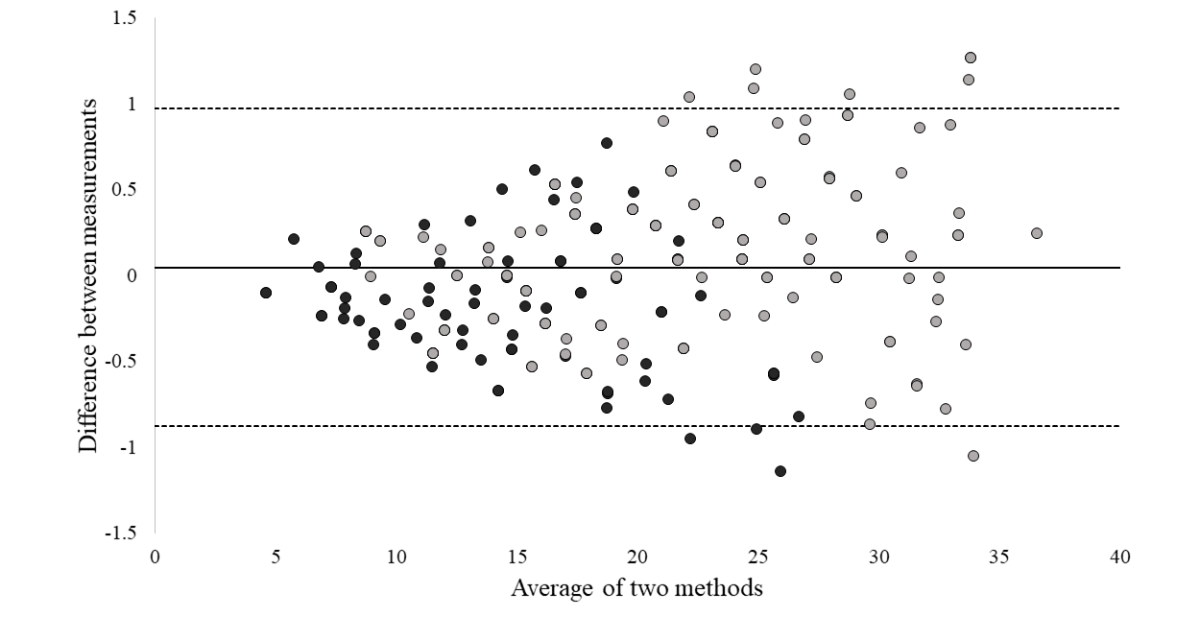
Bland-Altman plot of both measuring methods: countermovement jump depicted by dark gray points and countermovement jump with arm swing depicted by light gray points.

Correlation and Bland-Altman analyses were performed from data obtained and are shown in Figure 9 and Figure 10, respectively. Correlation analysis shows a coefficient of determination of *R*^2^=.996. These analyses demonstrate that the proposed system not only has a high correlation, but it shows that the difference of the two paired measurements is really low, which means that both methods produce systematically the same results.

**Figure 11:**
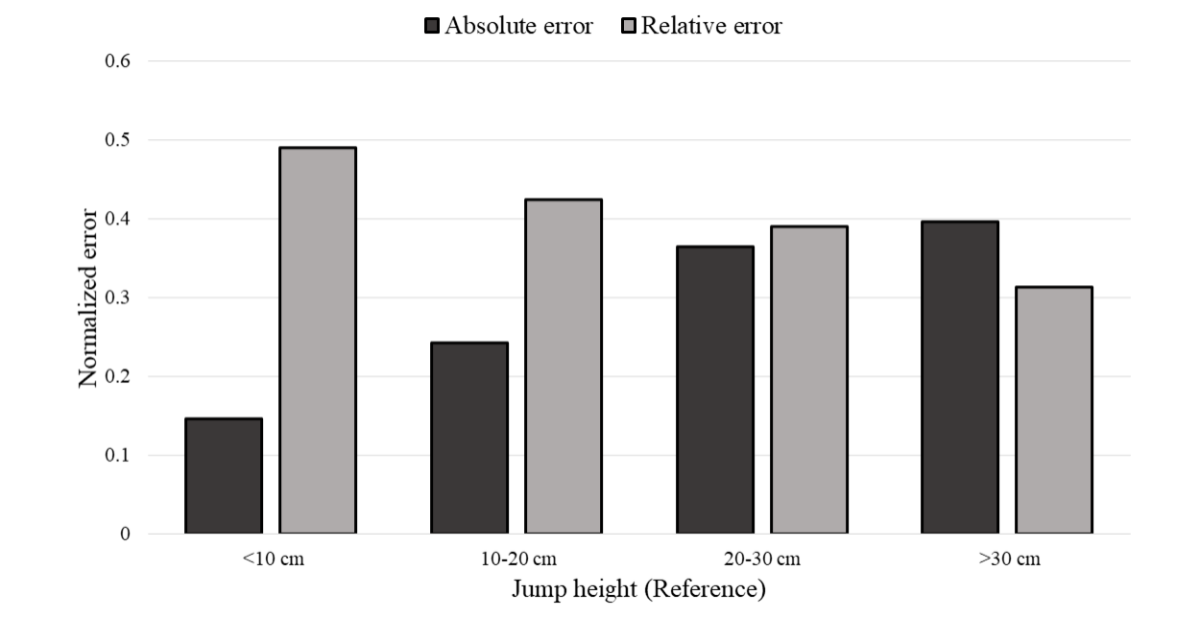
Normalized mean absolute error and mean relative error, divided in different ranges of height reached during vertical jump.

In Figure 11, the normalized MAE and MRE are shown for different ranges of jump heights reached. By analyzing the different ranges of height reached by the volunteers, which are <10 cm, 10 to 20 cm, 20 to 30 cm, and >30 cm, MREs obtained were 2.38%, 2.07%, 1.90%, and 1.54%, respectively. MAE obtained were 0.18 cm, 0.31 cm, 0.46 cm, and 0.50 cm, respectively. From this data, no significant difference can be found. However, it can be noticed that MAE increases as jump height increases, while MRE decreases.

**Figure 12:**
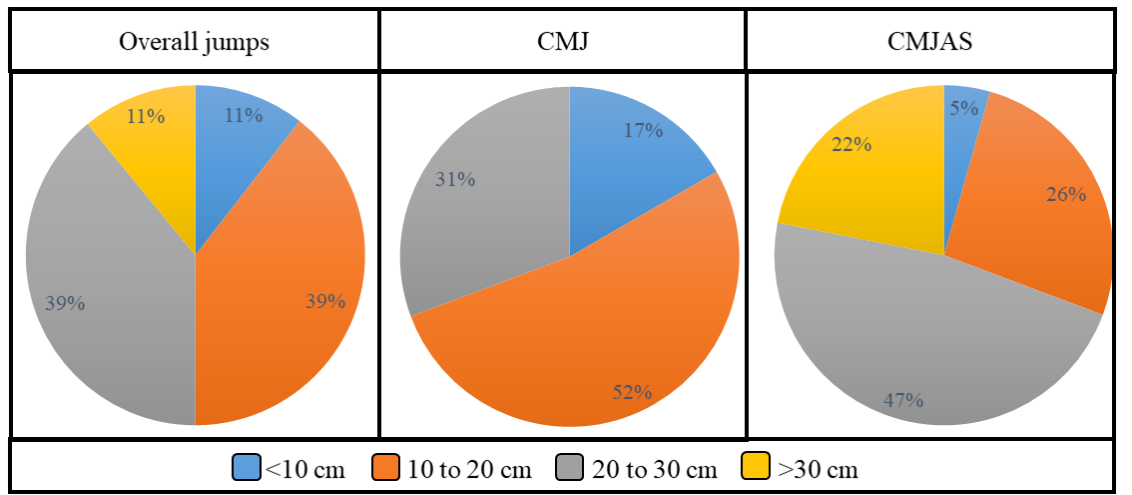
Distribution of different height ranges reached by the users. Overall trials, only countermovement jump trials, and only countermovement jump with arm swing trials are shown.

Figure 12 shows the charts with distribution of the heights reached by the volunteers when performing the jump trials. For CMJ, no volunteer was able to surpass the 30 cm height. However, by adding the arm swing, 22% of the volunteers surpassed the 30 cm height. Also, for CMJAS trials, 47% of the subjects reached a height ranging from 20 to 30 cm, compared with CMJ, in which only 31% of the subjects reached this height.

### Second Experiment: Sampling Frequencies Comparison

In this experiment, the effects of different sampling frequencies were analyzed. Raw data from the system was obtained for a total of 90 jumps (45 CMJs and 45 CMJASs). An offline emulation of different sampling frequencies was performed through downsampling of this raw data. This means samples are removed to emulate a slower sampling frequency. With this method, and with the base sampling period of 5 ms from the system, 200 Hz, 100 Hz, 66.6 Hz, 50 Hz, 40 Hz, 33.3 Hz, 28.5 Hz, 25 Hz, 22.2 Hz, and 20 Hz frequencies were emulated.

Similar to the first experiment, the error was calculated using the high-speed camera as reference. For this analysis, only MRE was obtained for each sampling frequency to assess which frequencies are able to maintain a relative error below 5%. Results show that sampling frequencies of 200 Hz, 100 Hz, and 66.6 Hz have similar performance, with relative errors of 1.88%, 2.22%, and 2.88%, respectively. However, the maximum error among the 90 trials increases considerably between these frequencies, with maximum errors of 5.27%, 7.02%, and 8.25% for each respective frequency. Sampling frequencies of 50 Hz, 40 Hz, and 33.3 Hz also show good performance regarding the relative error, which is maintained below 5% for the 3 cases, but the maximum relative error found in these 3 frequencies is considerably higher than the found in the previous set.

**Figure 13:**
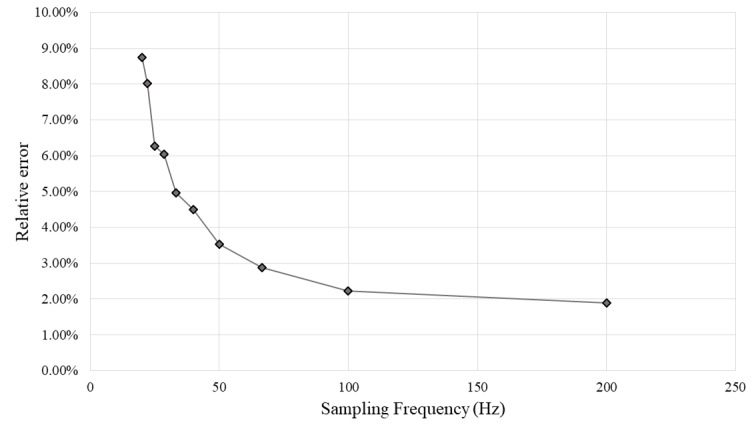
Mean relative error obtained for each proposed sampling frequency. As sampling frequency decreases, relative error increases exponentially.

In Table 4, MRE and maximum and minimum relative errors found among trials for the different sampling frequencies are shown. With slower sampling frequencies, MRE increases exponentially as shown in Figure 13, which suggests that sampling frequencies equal to or below 28.5 Hz are not reliable enough to maintain MRE below 5%. Also, sampling frequencies slower than 50 Hz and 33.3 Hz show maximum relative error among trials higher than 10% and 20%, respectively.

Table 5 shows how MRE is distributed in different ranges. Only 200 Hz and 100 Hz sampling frequencies are able to maintain 95% of their results within 5% of relative error. Also, sampling frequencies slower than 50 Hz considerably increase the percentage of relative errors found above 5%. These results suggest that sampling frequencies of 200 Hz and 100 Hz are the most reliable, frequencies of 66.6 Hz and 50 Hz have an acceptable performance, and the remaining sampling frequencies are unreliable for this specific application.

**Table 4:** Mean relative error and maximum and minimum relative errors obtained from all 90 trials for each sampling frequency analyzed.

Relative error	Sampling periods/frequencies									
	5 ms, 200 Hz	10 ms, 100 Hz	15 ms, 66.6 Hz	20 ms, 50 Hz	25 ms, 40 Hz	30 ms, 33.3 Hz	35 ms, 28.5 Hz	40 ms, 25 Hz	45 ms, 22.2 Hz	50 ms, 20 Hz
MRE^a^	1.88	2.22	2.88	3.52	4.50	4.97	6.04	6.27	8.02	8.75
MAX^b^	5.27	7.02	8.25	9.73	14.25	14.25	21.30	19.73	28.39	32.11
MIN^c^	0	0	0	0	0.70	0	0	0	0	0.73

^a^MRE: mean relative error.

^b^MAX: maximum relative error.

^c^MIN: minimum relative error.

**Table 5:** Percentage of trials whose relative error is within the ranges of 5% or less, higher than 5% and lower than 15%, and higher than 15%.

Relative error	Sampling periods/frequencies									
	5 ms, 200 Hz	10 ms, 100 Hz	15 ms, 66.6 Hz	20 ms, 50 Hz	25 ms, 40 Hz	30 ms, 33.3 Hz	35 ms, 28.5 Hz	40 ms, 25 Hz	45 ms, 22.2 Hz	50 ms, 20 Hz
RE^a^ ≤5%	98.89	94.44	87.78	76.67	58.89	57.78	48.89	52.22	35.56	27.78
RE 5% to 15%	1.11	5.56	12.22	23.33	41.11	42.22	47.78	43.33	54.44	57.78
RE >15%	0	0	0	0	0	0	3.33	4.44	10	14.44

^a^RE: relative error.

When MRE is obtained from different jump heights (≤10 cm, 10 to 20 cm, 20 to 30 cm, and >30 cm) at each sampling frequency, the relevance of a proper sampling frequency when calculating height reached for small jumps (<20 cm) is observed. When using sampling frequencies slower than 50 Hz, MRE obtained from these small jumps is always higher than 5%, and for the slowest sampling frequency, MRE reaches a value of 21.50% for jump heights smaller than 10 cm. For higher jump heights, an increase in MRE (>5%) is noticeable for sampling frequencies slower than 40 Hz, reaching a value of up to 9.15% for the slowest sampling frequency. A summary of these results is shown in Table 6.

**Table 6:** Mean relative error obtained from each of the analyzed sampling frequencies for different ranges of height reached during the vertical jump.

Jump height	Sampling periods/frequencies									
	5 ms, 200 Hz	10 ms, 100 Hz	15 ms, 66.6 Hz	20 ms, 50 Hz	25 ms, 40 Hz	30 ms, 33.3 Hz	35 ms, 28.5 Hz	40 ms, 25 Hz	45 ms, 22.2 Hz	50 ms, 20 Hz
<10 cm	1.31	2.29	4.33	2.62	5.28	5.09	5.40	6.29	10.77	21.50
10-20 cm	2.23	2.40	2.89	4.29	5.21	5.45	7.55	8.60	9.65	8.42
20-30 cm	1.91	2.31	3.02	3.55	4.62	5.29	5.06	6.12	6.96	9.15
>30 cm	1.60	1.95	2.50	2.92	3.63	4.17	5.83	4.34	7.32	6.85

## Discussion

### Principal Findings

The vertical jump is a test commonly used by health care professionals to assess strength in the lower limb muscles of a subject. Although this test is widely used for strength assessment among athletes, relevant information can be obtained from people with no relevant sports background.

An important point to highlight about the system developed in this study is its low price. The total for components used in construction is approximately US $40. In comparison with commercially available devices, this developed system is significantly more affordable. Among the devices commonly used on medical and sports fields to measure vertical jump height are the vertical jump test mat (Gill Athletics) [31], Just Jump system (Perform Better) [10,32], Vertec device (Gill Athletics) [10,33], electronic vertical jump tester (Gill Athletics) [34], Optojump testing (Perform Better) [12,35], and bilateral force plate (Hawkin Dynamics) [18,36]. Our proposed system will have to pass through different standards and certifications (like ISO standards [37]) before it can be considered as a standard medical device. Table 7 shows a comparison of prices between commercially available devices and the system proposed here. Prices of the commercially available devices are listed as found at the moment of writing this article.

**Table 7:** Comparison of prices between commercially available devices and the system developed in this study.

Device for vertical jump measurement	Price $
Proposal from this study (estimated price of components)	40
Vertical jump test mat (Gill Athletics) [31]	360
Just Jump System (Perform Better) [32]	629
Vertec device (Gill Athletics) [33]	760
Electronic vertical jump tester (Gill Athletics) [34]	2925
Optojump testing (Perform Better) [35]	3804
Bilateral force plate (Hawkin Dynamics) [36]	5000

Throughout data capturing in both experiments, some important points can be highlighted. Despite the advantages that the proposed system and reference device offer, both have an inherent error due to their sampling frequency (more specifically due to their sampling period). The proposed system has a sampling frequency of 200 Hz, and thus the sampling period is 5 ms. Likewise, the reference device has a sampling frequency of 120 Hz and a sampling period of 8.3 ms. This means that every sampling period each device updates its readings, which implies an uncertainty of the sampling period between data updates. In other words, there is an inherent uncertainty in the system during the takeoff and landing phases of the jump, time span that is used to calculate height reached. From both phases, the proposed system has a total uncertainty of 10 ms, while the reference device has a total uncertainty of 16.6 ms. This inherent error is characteristic of electronic devices and directly related to their sampling frequency. Nonelectronic methods for jump height measuring lack of this inherent error, but as stated before, these methods tend to overestimate obtained measurements and are less precise.

Regarding the high-speed camera used as a reference device, when the recorded videos were analyzed, the ease of selecting the correct frames depended on the correct technique execution of the volunteer: taking off from both forefeet at the same time during the takeoff phase and landing with both forefeet at the same time during the landing phase. This was the ideal technique execution. On the other hand, some volunteers either took off or landed with only one forefoot and not with the same foot in some occasions. In such cases, it was harder to select the takeoff and landing frames. This is difficult for volunteers to control without long-term training in the proper technique.

On the proposed protocol, the inclusion of two different jump techniques proved to be useful in order to increase the dynamic range of the data. The difference between the CMJ and CMJAS was significant. The addition of an arm swing increased jump height an average of 44.84% in the first experiment and 34.86% in the second experiment.

### Limitations

One of the main limitations of the developed system was its sampling frequency. Although the microcontroller used had a high CPU frequency, the sampling frequency was limited because of the number of operations needed for a full scan of the mat (16 rows and 16 columns, a scan of 256 individual cells) and number of operations this implies. Another limitation was the total sensing area of the system of 30×30 cm. Although no volunteer reported discomfort, the total area limits the stance of volunteers; in addition, the landing phase of every jump trial must be performed in a controlled manner, so the volunteer lands inside this area.

These limitations can be solved in future versions of the mat. The design of the mat can be modified to increase its total sensing area and the size of each row and column, so in this way with fewer number of rows and columns the same sensing area could be achieved, thus increasing the sampling frequency of the system. However, this would diminish the resolution of the system.

### Conclusions

In this study, a novel low-cost system for measurement of the jump height is proposed. Two experiments were performed—one to validate the system and the other to assess the effects of different sampling frequencies.

When evaluating the performance of the proposed system in the first experiment, results show that with the proposed sampling frequency of 200 Hz relative error for all of the 228 jump trials is maintained below 5%. In the second experiment, with sampling frequencies of 200 Hz and 100 Hz, relative error is maintained below 5% for 98.89% and 94.44% of the jump trials, respectively.

The flight-time formula is a widely used, validated method to calculate height reached during vertical jumps. A high-speed camera as reference device has been used in related studies along with the flight-time formula, proving to be a reliable tool. Our first experiment showed through correlation and Bland-Altman analyses that the proposed system and a high-speed camera reference device produced systematically similar results when calculating jump height.

Our second experiment concluded that 200 Hz and 100 Hz sampling frequencies have similar performance, and both frequencies are reliable when calculating jump height using the flight-time formula. This implies that if access to hardware capable of processing data at 200 Hz were limited, hardware capable of processing data to at least 100 Hz could offer similar results. However, if higher sampling frequencies are available, they should be used.

These results demonstrate that the proposed system is as reliable as a commercially available device, and the selected sampling frequency of 200 Hz is reliable for obtaining relative errors below 5% for at least 95% of the jump trials. The proposed system offers an alternative for health care professionals to use a mobile monitoring station of their choice, and its price is more affordable than commercially available devices.

## Abbreviations

CMJ: countermovement jump
CMJAS: countermovement jump with arm swing
FSR: force-sensitive resistor
MAE: mean absolute error
MRE: mean relative error
